# Dynamic measures for transportation networks

**DOI:** 10.1371/journal.pone.0242875

**Published:** 2020-12-03

**Authors:** Oriol Lordan, Jose M. Sallan

**Affiliations:** Department of Management, Universitat Politècnica de Catalunya, Terrassa, Catalunya, Spain; University of Hail, SAUDI ARABIA

## Abstract

Most complex network analyses of transportation systems use simplified static representations obtained from existing connections in a time horizon. In static representations, travel times, waiting times and compatibility of schedules are neglected, thus losing relevant information. To obtain a more accurate description of transportation networks, we use a dynamic representation that considers synced paths and that includes waiting times to compute shortest paths. We use the shortest paths to define dynamic network, node and edge measures to analyse the topology of transportation networks, comparable with measures obtained from static representations. We illustrate the application of these measures with a toy model and a real transportation network built from schedules of a low-cost carrier. Results show remarkable differences between measures of static and dynamic representations, demonstrating the limitations of the static representation to obtain accurate information of transportation networks.

## Introduction

Complex networks theory studies global properties of systems composed by a large quantity of interconnected elements. Modelling those systems as complex networks, where the elements are the nodes and the links the connections among them, we are able to gain insight into system’s structural properties, and to learn how those systems grow and evolve. Global structural properties are described using network measures: for instance, we can say that a network has the small world property if it has a large average clustering coefficient and a small average path length [1]. On the other hand, real-world complex networks are heterogeneous, meaning that not all nodes and edges are equally relevant or central [2, 3]. To account for this, we define parameters as degree for nodes (the number of connections to the node) and betweenness for nodes and edges (nodes and edges of high betweenness are frequently present in shortest paths) as centrality measures of network elements. Node and betweenness are the most frequent measures of centrality, although other measures have been defined in the literature [4, 5].

A relevant family of complex networks are transportation systems such as roads, rail and marine or air transport. In those systems, nodes are geographical locations, and links represent connections between these locations. Application of complex networks theory to transportation systems allows to identify relevant locations and connections of the system, and to gain insight on properties like network robustness [6–9], jamming transitions [10, 11] or epidemic spreading [12, 13].

When studying a transportation system, the predictions and insights obtained will be as good or accurate as the model we have used to analyse them. The most usual way to construct a transportation network is to collect information about existing connections on a time horizon, and consider a pair of nodes linked by an edge if there is at least a direct connection between them. In this *static representation*, two nodes are connected if there is at least a path between them. In spite of its extensive use in modelling complex transportation networks [7, 14–16], static representations can be too simplistic for some purposes, specially when computing and interpreting measures obtained from shortest paths among nodes, or evaluating connectivity between two nodes (i.e., assessing which pairs of nodes are connected by a path in the network). First, static representations are frequently unweighted. Unweighted connections use topological distance, meaning that distance between to nodes is equal to the smallest number of edges required to connect them, irrespective of time or distance. When using topological distance, two paths connecting a pair of nodes with the same number of edges are considered of equal length, although reaching the destination may take longer for one of the paths. Second, most collective means of transportation use scheduled connections to aggregate travel demand. Every schedule is defined by departure and arrival times, and offers possibilities of indirect connectivity, as travellers take two or more connections to get to their destination. When building the static representation, information about schedules is lost: as a consequence, some of the possibilities of connection of static representations may not exist, as connecting schedules are not synced. This might mean that two nodes that appear connected in the static representation are not really connected. Third, time travel of a connection between two nodes may not be constant, due to traffic congestion or unexpected incidents.

The shortcomings of the static, unweighted representation of transportation systems can be overcome with more precise representations. We can achieve this by relying on temporal networks theory, which adds the moment when interactions take place to the set of elements and interactions to model complex systems [17]. Temporal networks theory allows a more precise modelling of human communications [18], human and animal proximity networks, distributed computing, citation networks and brain networks, among others (see [19] for a review). Many of these systems are contact networks, where indirect connections are carried along time-respecting paths. A path A-B-C exists if A and B contact prior to B and C. Temporal networks theory may help to build a more precise representation of transportation networks, but differences between contact and transportation networks must be taken into account. First, we can define shortest paths using temporal distance: the shortest path between two nodes will be the one that connects them in the shortest time, irrespective of the number of intermediate connections needed [20]. Temporal distance allows a definition of weighted transportation networks where the edge weight is the amount of time needed to make the connection. Note that in these weighted networks we are assigning time travel to edges, rather than intensity or frequency of connections, like in [21]. Second, when defining shortest paths in transportation networks we must take into account travel times and waiting times, unlike in contact networks. In transportation networks, shortest paths depend on schedules available at a specific time, so that temporal distance and the shortest path itself may vary along the time horizon considered, so that for any pair of nodes a temporal distance profile is considered [22]. Finally, contact networks can be much more dynamic than transportation networks, in the sense that in contact networks topology can change rapidly [23], and new nodes and edges can be added along the time horizon. [24]. This dynamic behaviour is unlikely to happen in transportation networks, as long as there are no incidents affecting the infrastructure (for instance, removal of a link because an accident). It is more frequent, though, that time travel of specific connections experiences variations along the time horizon because of accidents or traffic congestion. The calculation of shortest paths in that situation is more complex [25], leading also to a temporal distance profile, even if there are no scheduled connections.

The inclusion of information about the time of interaction, and the computation of time-respecting paths between network nodes has allowed the definition of measures for temporal networks, although most of them have been developed for contact networks [23, 26, 27]. The aim of our paper is to define a set of dynamic measures that take into account the specificities of transportation networks. By doing this, we contribute to complex networks theory and transportation science: we enrich temporal networks theory by considering specificities of transportation networks, and transportation science by allowing a more precise evaluation of importance and centrality of nodes and edges, and of overall performance of the transportation system. The rest of the paper is organised as follows: first, we define the static and dynamic representations of transportation networks, which in turn can be weighted and unweighted, and define dynamic shortest paths. In the next section, we use the definition of shortest paths to define network, node and edge measures for dynamic representations of transportation networks. In the materials and methods section, we describe how do we compute dynamic shortest paths, and the characteristics of our case study, a transportation network defined from a sample of schedules of a low cost airline. In the applications section, we introduce the measures for each representation with a toy model, and then we compare the static and dynamic measures our case study, which allows us to illustrate how dynamic measures provide a more precise evaluation of the transportation network. The results are summarised in the conclusions section.

## Static and dynamic representations of transportation networks

The most usual representation of transportation networks is *static*. In a static representation, a connection *i* → *j* exists if there is a direct connection between *i* and *j* during a time horizon. Indirect connections are considered if a path can be established between a pair of nodes. This representation can be unrealistic, as some of the paths defining indirect connections may be constructed with non-synced schedules. A way to avoid this pitfall is to build a *dynamic* representation considering all existing connections with compatible schedules in the time horizon. In this representation, an indirect connections between two nodes exists if there is a temporal path of synced schedules connecting them. In *unweighted* graphs, the path length of an indirect connection is equal to its number of connections. For instance, a route of two intercontinental flights has the same distance as a route of two regional flights. As effectiveness of transportation networks is associated with fast connections, it can be useful to determine the time spent to reach node *j* from node *i*. This can be achieved defining *weighted* graphs, assigning weights to edges equal to time travel. In *weighted* graphs, the path length of a indirect connection is equal to the sum of edge weights of the path, that is, the total travel time of the connections. When we consider edge weights, we define *dynamic weighted* measures, and *dynamic unweighted* measures otherwise. The definition of distances for static representations is straightforward. Static unweighted distance is equal to the smallest number of edges connecting nodes *i* and *j*, and static weighted distance is the minimum value of sum of weights of edges connecting a pair of nodes.

For dynamic representations, distances can be defined through dynamic shortest paths [25]. The *temporal distance* *τ*_*ij*_(*t*) is the minimum time to reach *j* from *i* along temporal paths at time *t*. As temporal distance depends on available schedules at time *t*, we have a temporal distance profile for the time horizon, defined by the value of temporal distances for each *t* [22]. If we can choose the moment we depart from an interval [*t*_*s*_, *t*_*f*_], with *t*_*s*_, *t*_*f*_ ∈ [0, *T*] we can define *the dynamic weighted distance* between *i* and *j* as:

$$\tau_{ij}^{*}=\underset{t\in [t_{s},t_{f}]}{\text{min}\;}\tau_{ij}(t)$$

To obtain the *number of dynamic shortest paths* $\sigma_{ij}^{D}$, we retain only the paths that connect *i* and *j* with time $\tau_{ij}^{*}$ using the minimal number of steps. Making this choice, we take into account user’s preferences for routes of shorter travel time, shorter connection times and fewer connections [28]. This value of minimum schedules $s_{ij}^{*}$ is the *dynamic unweighted distance* between *i* and *j*. So in a dynamic representation of a transportation network we can obtain for each pair of nodes (*i*, *j*):

1. The dynamic weighted distance $\tau_{ij}^{*}$
2. The dynamic unweighted distance $s_{ij}^{*}$
3. The number of dynamic shortest paths $\sigma_{ij}^{D}$

## Dynamic measures of transportation networks

### Network measures

There are three widely used network measures based on shortest paths: characteristic path length, diameter and efficiency. *Characteristic path length* *L* is equal to the average value of non-divergent (non-infinite) distances. Two dynamic measures of *L* can be obtained for unweighted *U* and weighted *W* graphs:

$$L^{DU}=\frac{1}{P^{D}}\sum_{i\neq j,s_{ij}^{*}<\infty }s_{ij}^{*}\;L^{DW}=\frac{1}{P^{D}}\sum_{i\neq j,\tau_{ij}^{*}<\infty }\tau_{ij}^{*}$$

where *P*^*D*^ is the number of non-divergent dynamic paths. Accordingly, we have also two values for *network diameter*, the maximum value of non-divergent topological and temporal distances, respectively.

*Network efficiency* is equal to the harmonic mean of distances between nodes [29]. As this measure sets contributions of all non-divergent paths equal to zero, does not diverge for unconnected graphs as characteristic path length does. Efficiency measures of a network with *N* nodes can be computed as follows:

$$E^{DU}=\frac{1}{N(N-1)}\sum_{i\neq j}\frac{1}{s_{ij}^{*}}\;E^{DW}=\frac{1}{N(N-1)}\sum_{i\neq j}\frac{1}{\tau_{ij}^{*}}$$

### Node measures

There are two relevant node measures based on shortest paths in dynamic representations: betweenness and closenness [5]. Other measures of node centrality not based on shortest paths, such as node degree, make sense only on static representations [17]. *Node betweenness* is an important measure in transportation networks. Nodes with high betweenness represent *central* nodes, as they are in the middle of many scheduled connections [30], and therefore are topologically important nodes in the network. Betweenness is defined using the number of shortest paths *σ*_*jk*_ between *j* and *k*, and the number of shortest paths between *j* and *k* that pass through node *i* *σ*_*jk*_(*i*). In the dynamic representation only the existing paths that connect every pair of nodes in minimum time, taking the minimal number of steps are considered. Therefore, *dynamic node betweenness* can be defined as:

$$b_{i}^{D}=\sum_{j,k\in N,j\neq k}\frac{\sigma_{jk}^{D}(i)}{\sigma_{jk}^{D}}$$

*Closeness* of node *i* is defined as the inverse of the sum of distances from *i* to all other *k* nodes. We can define two dynamic closeness measures, unweighted and weighted,:

$$c_{i}^{DU}=\frac{1}{\sum_{j\in N}s_{ij}^{*}}\;c_{i}^{DW}=\frac{1}{\sum_{j\in N}\tau_{ij}^{*}}$$

If any of the elements of the distance matrix is divergent (i.e., the corresponding pair of nodes is not connected) the definition of closeness is problematic. This can be solved using *harmonic centrality*, defined as:

$$h_{i}^{DU}=\sum_{j\in N}\frac{1}{s_{ij}^{*}}\;h_{i}^{DW}=\sum_{j\in N}\frac{1}{\tau_{ij}^{*}}$$

Harmonic centrality is strongly correlated with closeness [5], but naturally accounts for nodes that cannot reach *i*, so it is more suitable for graphs that are not strongly connected [31].

### Edge measures

For dynamic representations, a *dynamic edge betweenness* for a direct connection *ℓ* between a pair of nodes can be defined as:

$$\begin{array}{c} b_{\ell }^{D}=\sum_{j,k\in N,j\neq k}\frac{\sigma_{jk}^{D}(\ell )}{\sigma_{jk}^{D}} \end{array}$$

where $\sigma_{jk}^{D}(\ell )$ is the number of dynamic shortest paths between *j* and *k* that use edge *ℓ*.

## Materials and methods

### Computation of dynamic shortest paths

For computing dynamic measures, we use a set of scheduled connections to build a time-node network. In this temporal network representation, there is a node for each combinations of arrivals and departures destinations and times. This node has one node for each departure or arrival, defined by a the location of each event and its timestamp. To account for available connections in a location (e.g., connecting flights in an airport), we have considered a minimum connecting time. To compute the dynamic measures, we have obtained the paths of minimal duration for the resulting network. Then, we have collapsed these paths for each location to obtain the shortest paths between locations. We have retained the quickest paths that connect each pair of locations with minimal steps.

### Application to a low-cost airline

We illustrate the computation of dynamic measures in real transportation networks with the flights scheduled by Ryanair departing from local times 06:00 AM of August 1st, 2015 to 05:59 AM of August 2nd, 2015. In this time horizon, the airline has scheduled 1,746 flights, of which 20 are non-returning flights, probably because of missing data. To obtain an undirected static representation, we have not considered the later, so the resulting network includes 1,726 legs, leading to a static representation with 695 bidirectional connections between 158 airports. Defining the dynamic representation for a transportation network requires defining some constraints for considering shortest paths. First, we need to consider that passengers have constraints to transfer between flights. Although low-cost carriers operate on a point-to-point basis, it is frequent that passengers arrange self-hubbing connections purchasing one ticket for each flight [32]. Similarly to previous research on self-hubbing [33], we decided to allow transfer times equal or larger than 60 minutes. For this example, no maximum transfer time was considered.

## Applications

We apply the above definitions to a toy model, and to the real air transport network of a low-cost carrier.

### Toy model

**Fig 1:**
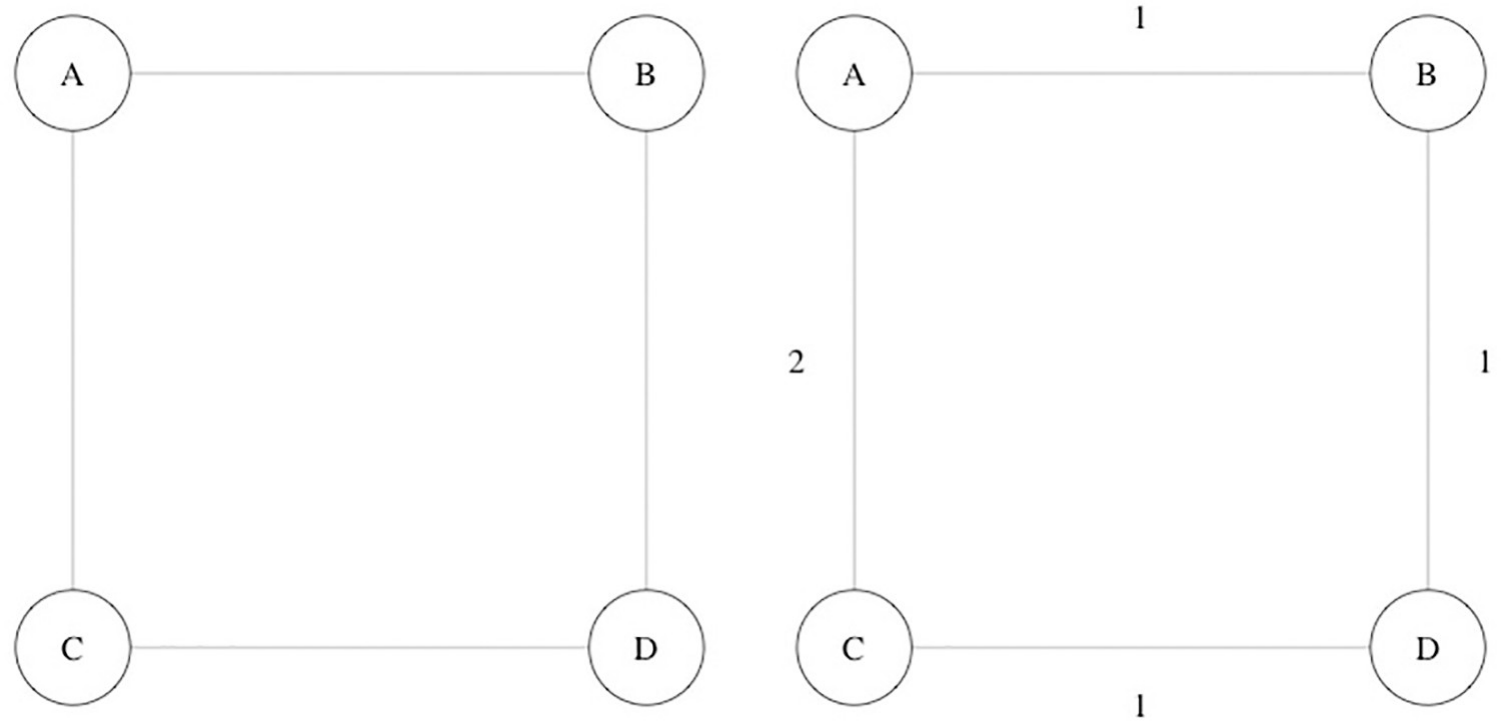
Toy model: Unweighted (left) and weighted (right) graphs for the static representations.

In order to explain the above definitions, we propose a toy model with four nodes, with the connections defined in Table 1. The static representations, unweighted and weighted, of the toy model can be seen in Fig 1.

**Table 1:** A set of schedules between four nodes.

Departure	Arrival	Departure time	Arrival time
*A*	*B*	7	8
*A*	*C*	0	2
*B*	*A*	3	4
*B*	*D*	3	4
*C*	*A*	5	7
*C*	*D*	2	3
*D*	*B*	0	1
*D*	*C*	4	5

For this model, unweighted distances are equal in dynamic and static representations, but weighted distances are not. In Table 2 are presented the static and dynamic measures of closeness. In this case, we can compare closeness measures, as there is either a direct or indirect connection between any pair of nodes in both representations. Matrices **D**^*SU*^, **D**^*SW*^, **D**^*DU*^ and **D**^*DW*^ show the unweighted (U) and weighted (W) distances between nodes for the static (S) and dynamic (D) representation (note that the dynamic weighted distance matrix **D**^*DW*^ is not symmetric). Table 3 presents the values of network measures for each representation.

$$\begin{array}{cccc} {\text{D}}^{SU} & =(\begin{array}{cccc} - & 1 & 1 & 2\\ 1 & - & 2 & 1\\ 1 & 2 & - & 1\\ 2 & 1 & 1 & - \end{array}) & {\text{D}}^{SW} & =(\begin{array}{cccc} - & 1 & 2 & 2\\ 1 & - & 2 & 1\\ 2 & 2 & - & 1\\ 2 & 1 & 1 & - \end{array})\\ {\text{D}}^{DU} & =(\begin{array}{cccc} - & 1 & 1 & 2\\ 1 & - & 2 & 1\\ 1 & 2 & - & 1\\ 2 & 1 & 1 & - \end{array}) & {\text{D}}^{DW} & =(\begin{array}{cccc} - & 1 & 2 & 3\\ 1 & - & 2 & 1\\ 2 & 3 & - & 1\\ 3 & 1 & 1 & - \end{array}) \end{array}$$

**Table 2:** Toy model: Node closeness and betweenness.

	Closeness				Betweenness		
	*c*^*SU*^	*c*^*SW*^	*c*^*DU*^	*c*^*DW*^	*b*^*SU*^	*b*^*SW*^	*b*^*D*^
*A*	14	15	14	16	1	0	1
*B*	14	14	14	14	1	2	0
*C*	14	15	14	16	1	0	2
*D*	14	14	14	15	1	2	1

**Table 3:** Toy model: Network measures.

	Unweighted			Weighted		
	*L*	*D*	*E*	*L*	*D*	*E*
Static	1.33	2	0.83	1.5	2	0.75
Dynamic	1.33	2	0.83	1.66	3	0.72

### A sample of a low-cost airline

We illustrate the computation of the defined dynamic transportation measures with a sample of flights of a low-cost airline (see Methods for the details). The picture of the connectivity using dynamic shortest paths is quite different than in the static representation. While in the later all connections between airports were available, in the former existed only 49.86% of possible (direct or indirect) connections. Then, the dynamic representation is directed and not strongly connected. Table 4 shows the values of network metrics for the static and dynamic representations of the network. The unweighted value of *L* is slightly smaller in the dynamic representation, as many of the long connections in the static representation are not present in the dynamic representation. If we exclude the connections not present in the dynamic representation to compute *L* in the static representation we obtain a value of *L* = 2.0135, smaller than the dynamic value for the same collection of routes. As for the rest of values, the dynamic values show equal or less connectivity than the static. Therefore, for this network the static representation overestimates efficiency, and underestimates weighted average shortest path length and diameter, as it only considers synced connections and takes into account waiting times between connections.

**Table 4:** Network measures for the low-cost carrier network (weighted measures in minutes).

	Unweighted			Weighted		
	*L*	*D*	*E*	*L*	*D*	*E*
Static	2.341	5	0.465	286	705	3.922e−3
Dynamic	2.295	5	0.246	518	1080	1.386e−3

**Fig 2:**
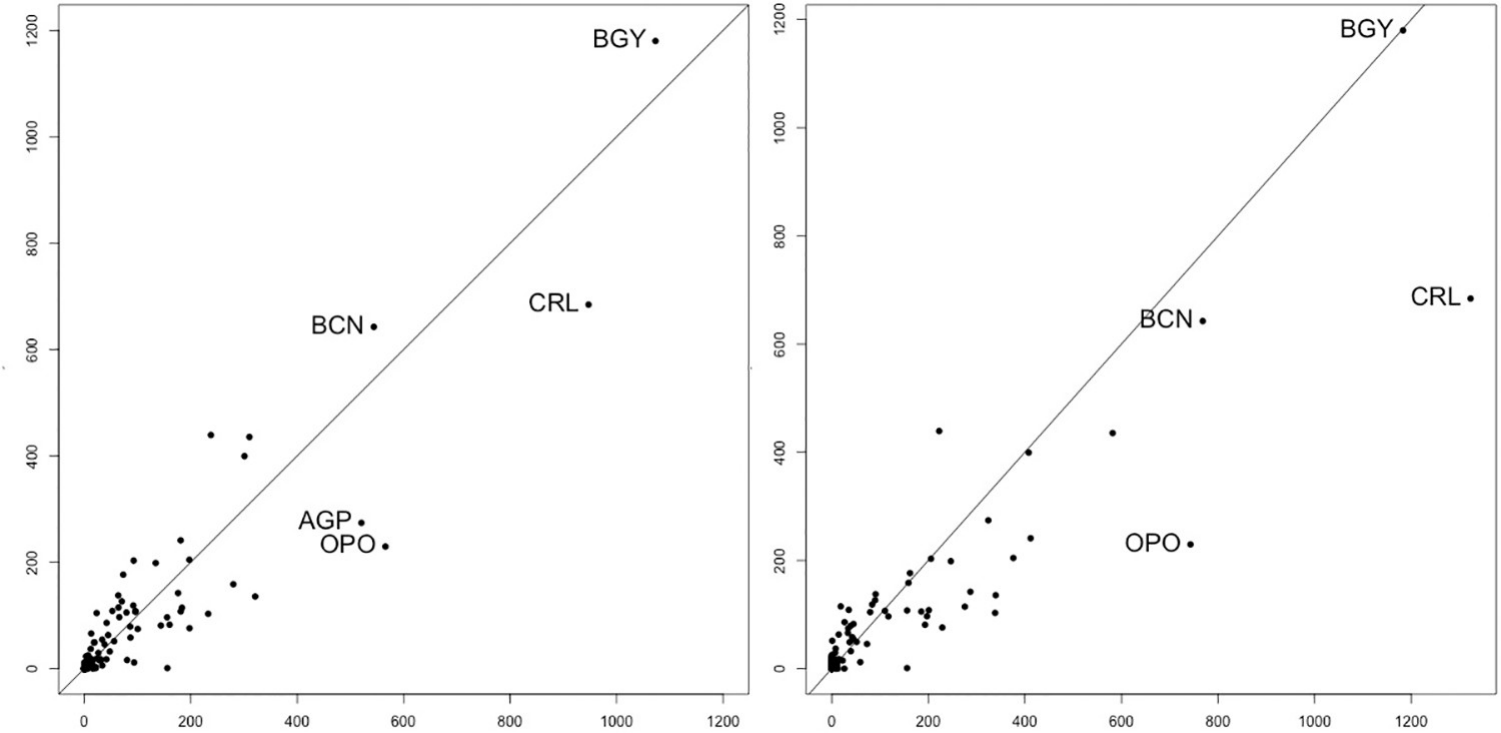
Values of betweenness obtained through dynamic representation vs. betweenness obtained through unweighted (left) and weighted (right) distances for the low-cost airline network (the most central airports STN and DUB have been omitted). The values of SU, SW and D for STN are 5163, 5646 and 4561. The values for DUB are 2212, 1644 and 1990.

In Fig 2 the values of dynamic betweenness are compared to static unweighted (left) and static weighted (rigth) betweenness. Two effects can explain differences between static and dynamic measures of betweenness. On the one hand, not all unweighted shortest paths take the same time, then some paths considered in static betweenness are not included in the dynamic betweenness. This effect can lead to higher or lower values of weighted and dynamic betweenness, compared with static betweenness. On the other hand, discarding unsynced connections makes many dynamic shortest paths between two nodes take more steps, so more nodes are intermediary in dynamic shortest paths. This effect increases the dynamic betweenness of the new intermediary nodes.

**Fig 3:**
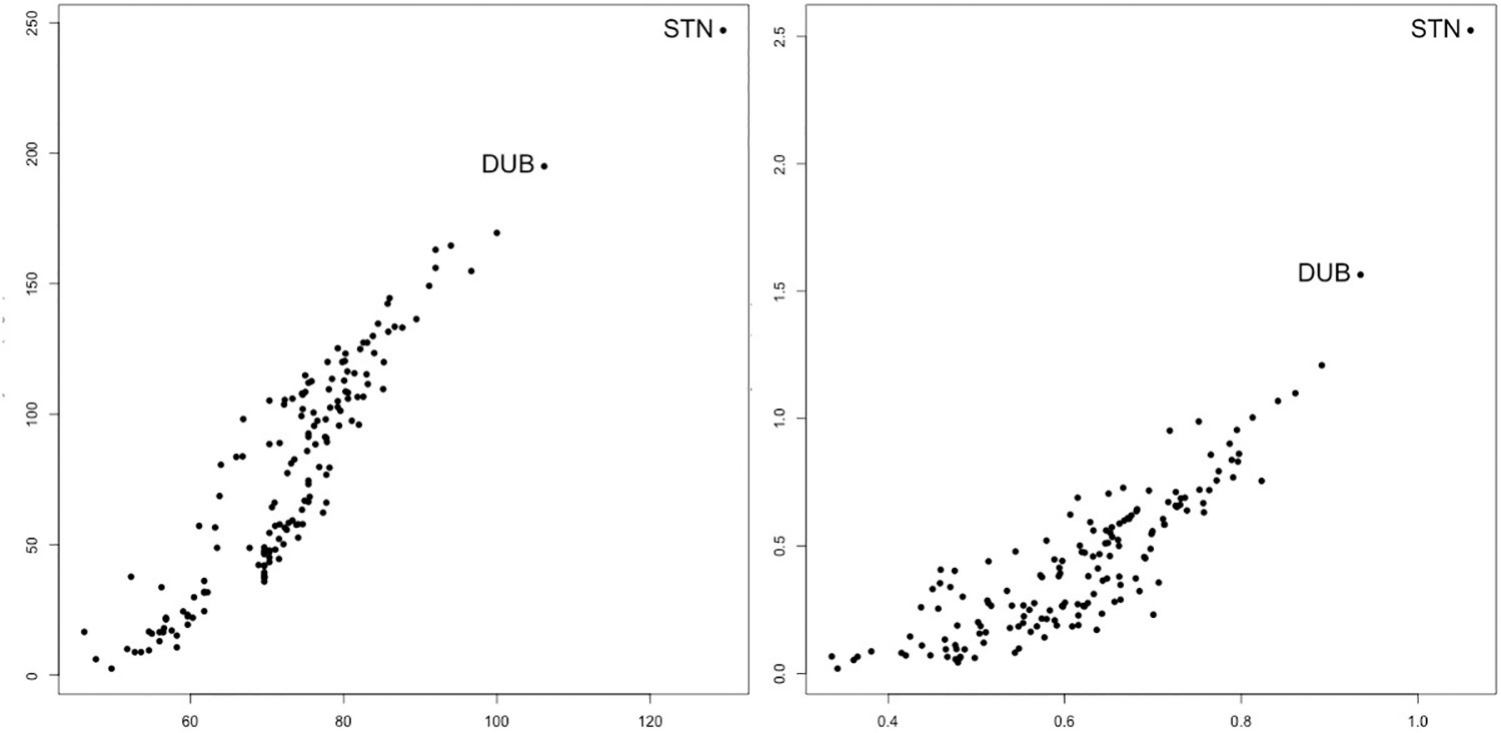
Comparison of static and dynamic values of harmonic closeness measured in steps or edges (left) and in time (right) for the low-cost airline network.

Fig 3 compares static and dynamic harmonic closeness with distances measured in steps (number of edges) and time. We have used harmonic closeness, as roughly a half of dynamic shortest paths are divergent. The airports with highest values of closeness are London-Stansted (STN), Dublin (DUB), Bergamo-Orio al Serio (BGY), Brussels South Charleroi (CRL) and Barcelona-El Prat (BCN). The five first airport of the ranking are the same for the edges-static harmonic closeness, and for the time static the fourth and fifth airports are Madrid-Barajas (MAD) and Roma-Ciampino (CIA). Airports with high values of harmonic closeness are the central airports of the network (e.g., STN, DUB) or airports with a central geographic position (CRL).

Finally, in Table 5 are listed the ten routes with highest values of unweighted, weighted and dynamic edge betweenness. We can observe that routes with high edge betweenness are between central airports, with the exception of the unweighted static representation, where the route with highest betweenness is between London-Stansted (STN) and Paphos International Airport (PFO). We can conclude that the high betweenness of this route in the static representation comes from considering connections between Greek airports that are unsynced with the existing scheduled connections.

**Table 5:** Routes of highest value of dynamic edge betweenness for the low-cost airline network.

unweighted		weighted		dynamic	
route	value	route	value	route	value
STN-PFO	254.552	DUB-STN	529.500	STN-DUB	590.667
DUB-STN	236.195	DUB-LTN	284	DUB-STN	337.667
STN-OPO	225.414	DUB-LPL	236.500	DUB-LPL	149.667
BGY-STN	203.034	DUB-MAN	212	BCN-STN	128
AGP-STN	174.980	ATH-CIA	202.667	STN-PMI	128
STN-MRS	174.360	DUB-CRL	191	STN-OPO	123.500
STN-PMO	161.429	ATH-PFO	186.333	LPL-DUB	122
PFO-GPA	157	STN-OPO	182	DUB-CRL	117.417
AGP-NUE	157	MAD-OPO	177.833	PSA-STN	116
DUB-BSL	157	DUB-BVA	170	STN-LEJ	116

## Conclusions

The most common way of modelling transportation networks in the literature is with a *static representation*: two nodes are connected by an edge if there is at least a direct connection between them in a specific time horizon. Time can be introduced in these models assigning weights to edges equal to the (minimal or average) time spent in a direct connection. The resulting weighted and unweighted static representations do not eliminate unsynced schedules or consider waiting times, so they are simplified representations of a transportation system.

A more realistic modelling of transportation networks can be implemented using *dynamic* representations, which include waiting times between connections and considers only temporal paths with synced connections. Dynamic representations allow the calculation of dynamic shortest paths. These are obtained retaining the temporal paths that link each pair of airports in minimum time, with the minimal number of steps.

Dynamic shortest paths allow the definition of more realistic versions of network measures, based on characteristic path length, diameter and efficiency measures defined for static, unweighted complex networks. Similarly, node betweenness- and closeness-based measures are defined. There is a single measure of dynamic betweenness, based on dynamic shortest paths. This definition can also be extended to edge betweenness.

After illustrating these measures with a toy model, in the Applications section we have applied the defined measures to a sample of scheduled connections of a low-cost airline, considering a 24 hour time horizon. Dynamic measures depict a more realistic description of network behaviour. Additionally, the comparison of results obtained for different graphs and representations allow us to gain insight into network properties. The dynamic representation is a realistic definition of transportation networks as, and therefore can help to better understand the behaviour of these networks facing phenomena such as delay propagation, node vulnerability or cascading failures, among others.
